# Potential Impact of Construction Noise on Selected Zoo Animals

**DOI:** 10.3390/ani9080504

**Published:** 2019-07-31

**Authors:** Richard Jakob-Hoff, Michael Kingan, Chiaki Fenemore, Gian Schmid, John F. Cockrem, Amanda Crackle, Emily Van Bemmel, Rebecca Connor, Kris Descovich

**Affiliations:** 1 New Zealand Centre for Conservation Medicine, Auckland Zoo, Auckland 1022, New Zealand; 2 Department of Mechanical Engineering, University of Auckland, Auckland 1010, New Zealand; 3 School of Veterinary Science, Massey University, Palmerston North 4442, New Zealand; 4 School of Environmental and Animal Sciences, Unitec Institute of Technology, Auckland 1142, New Zealand; 5 Centre for Animal Welfare and Ethics, School of Veterinary Science, University of Queensland, Gatton, QLD 4343, Australia

**Keywords:** zoo, behavior, welfare, noise, environment, elephant, giraffe, emu, alligator

## Abstract

**Simple Summary:**

Animals in zoos can adapt to many noises they hear on a regular basis. However, construction noise that is intense or occurs unpredictably may negatively impact the welfare state of some animals and induce a chronic stress response. This study aimed to understand the behavioral response to construction noise of selected species in an urban zoo in order to guide mitigating actions in advance of, and during, a planned construction project. The behavior of elephants, giraffes, emus and alligators was recorded during 90-min exposures to different sound environments including ambient sound, and four construction sound treatments. A non-invasive measure of physiological stress response was also measured in emus. All species appeared to respond to the recorded noise, with giraffes, elephants and emu, demonstrating behavioral changes potentially indicative of agitation or stress. This study has implications for the trade-offs that occur when zoos seek to improve long-term animal welfare through enclosure refurbishment and short-term impacts on animals exposed to construction noise.

**Abstract:**

In anticipation of a major construction project in an urban New Zealand zoo, a study was initiated to assess the response to construction noise of selected animal species (elephant, giraffe, emu and alligator) previously observed to be sensitive to this kind of noise. The overall aim was to detect any signs of aversive responses to this noise to enable keepers to recognize these and take any necessary mitigating actions during the construction period. The experimental approach involved the creation of acoustic maps of each focal animal enclosure, a series of 90-min video recordings of the animals’ behavior in response to ambient noise (control) and amplified broadcast of pre-recorded continuous and intermittent construction noise. Concentration of fecal corticosterone metabolites was also measured for the emus. Key findings were that giraffes, elephants and emus appeared to show an increase in behaviors that could indicate stress or agitation including vigilance and locomotion and may prefer quieter regions of their enclosure during sound exposure. Giraffes also increased close contact with conspecifics when exposed to construction noise. While alligators did not show clear evidence of noise-related stress, our findings indicated that all focal species showed some behavioral responses to recorded construction noise.

## 1. Introduction

The welfare of animals in their care is a key priority for zoos. The World Zoo and Aquarium Association promotes the ‘Five Domains’ animal welfare framework [1,2] as the gold standard against which to assess the welfare state of captive wildlife [3]. This framework comprises four measurable physical domains (nutrition, health, behavior and environment) that are collectively used to assess the affective state of animals as either negative, neutral or positive [2]. Implementation of this framework involves evaluation and ongoing review of husbandry and environmental conditions against current best zoo practice taking into account knowledge of the species’ biology in nature while recognizing that the needs of individuals may be diverse.

Left unmanaged, various aspects of a zoo environment may negatively impact the welfare state of captive animals [4] and, increasingly, the impact of these variables is being empirically studied [5,6,7,8,9]. One potentially significant stressor is the sound environment. Animals in zoos are routinely exposed to a variety of noises arising from visitors, ground maintenance, traffic and other sources. From time to time, zoo animals can also be exposed to potentially intense noise arising from construction activities [10,11,12,13]. Previous studies have demonstrated that this noise can elicit stress responses in some animals [12,13,14]. Exposure to stressors stimulates the release of adrenalin through the sympatho-adrenal-medullary system [15] and glucocorticoids via the hypothalamic–pituitary–adrenal axis (HPA) mobilizing glucose to increase available energy in order to respond to the potential threat [4,11,16,17,18]. Prolonged exposure can induce a state of chronic stress that can have deleterious effects on reproduction, immune status, growth and increased sensitivity to acute stress [4,19,20,21].

Zoo animals are exposed to both ambient sound conditions, and acute sound events, and both have potential to contribute to overall welfare states. Two large urban zoos in the USA measured an average sound pressure level of 70 dB while a Brazilian zoo found that the equivalent A-weighted continuous sound pressure (Leq) rose from 46.75 dB in the absence of visitors to 60.42 dB on days when the public were present [20]. These numbers exceed the 27–40 dB levels recorded in rainforest habitats and 20–36 dB in savannah habitats [21], suggesting that animals in zoos are exposed to sound levels exceeding those to which their species is naturally adapted. The US Department of Housing and Urban development considers Leq levels above 76 dB to be unacceptable outdoor noise levels in residential areas [22].

Several zoo-based studies have described aversive responses to construction or machinery noise in species such as snow leopards (*Panthera uncia*) [13], giant pandas (*Ailuropoda melanoleuca*) [12] and Hawaiian honeycreepers (*Drepanidinae* spp.) [14]. Owen et al. [11], studied the impact of ambient noise on captive giant pandas over a four-year period and found that behavioral agitation occurred more often on noisy days and some stress indices were more pronounced in the female during estrus and lactation [11]. Another zoo study [10] comparing the behaviors of a female sun bear (*Helarctos malayanus*) and her cub on the loudest (Leq 68.5 dB) and quietest (Leq 62 dB) days during the six month post-partum period found that the dam spent more time on cub-directed behavior and less time feeding on the louder days.

Auckland Zoo, the site of the current study, is located centrally in New Zealand’s largest city and, during the study period, housed approximately 1200 animals of 130 species including fish and invertebrates. In line with global trends, the zoo has progressively been modernizing its animal enclosures, public spaces and services and was scheduled to undertake its largest construction project to date during the period 2018–2020. This study was undertaken over the 12 months prior (beginning February 2017), in order to provide a scientific basis on which to make decisions to mitigate any negative impacts of construction noise on resident animals. Specific research questions were:

1. To what extent does exposure to construction noise affect activity budgets of focal zoo species?
2. Is behavior affected differently by continuous or intermittent noise?
3. Is exposure to construction noise associated with a significant increase in glucocorticoid concentration?
4. Do focal animals move away from the noise source during exposure?
5. Does noise exposure affect social spacing between individuals?

## 2. Materials and Methods

### 2.1. Experimental Approach

Given practical constraints imposed by working in a zoo environment, we elected to focus on specific behavioral changes between ambient and construction noise exposure to gain insight into the animals’ affective state. Where practically feasible, behavioral observations were supplemented with a measure of HPA axis activity [1,2]. Multiple individual, social, environmental and species-specific factors can influence the observed behavior of animals. Behavioral manifestations of stress are highly varied and can include increased vigilance (e.g., active monitoring of the environment), agonism (e.g., social conflict), stereotypies (e.g., repetitive, abnormal behaviors) and even increased lethargy (e.g., a behavior response that is abnormally decreased) [12,13,23,24,25]. In the current study, an experimental approach was taken to isolate, as far as possible, construction noise as the primary influence on observed behavior and physiological changes relative to those recorded under ambient sound conditions.

### 2.2. Animals and Facility

Nine animals of four species (Asian elephant, *Elephas maximus*, giraffe, *Giraffa camelopardalis*), emu, *Dromaius novaehollandiae* and American alligator, *Alligator mississippiensis)* were selected as focal subjects for this project (Table 1). Species selection was based on keeper observations of sensitivity to unusual noises, animal records, geographic proximity to the prospective construction site and known audio-sensory capabilities. In relation to the latter, West (1985) [26] reports that the lower and upper limits of hearing at a sound pressure level of 60 dB for an elephant (*Elephas maximus*) are, respectively, 17 Hz and 10.5 kHz (c.f. 29 Hz and 19 kHz for humans reported in the same study). Wever (1971) [27] reports that alligators (*Alligator mississippiensis*) exhibit a good level of “auditory capability” over the range 20 Hz–6 kHz with the best sensitivity in the region 150 Hz–3 kHz. There appears to be relatively little research on the hearing ranges of emu and giraffe. Manley et al. (1997) [28] report that the sensitive hearing range for emu chicks is from 50 Hz to 4 kHz which, according to Corfield et al. (2013) [29], covers the known range of their adult vocal communication signals. We were unable to uncover any reported hearing range for giraffe although Baotic et al. (2015) [30] report that giraffe do produce low frequency humming sounds with an average fundamental frequency of 92 Hz which “might be of communicative relevance”, suggesting that they are responsive to sounds at these frequencies.

All focal animals had been resident at the zoo since birth or for a minimum of 15 months. All of the animals were female with the exception of one emu, and all were adult except for one giraffe calf who was 2 months old at the commencement of the study. The elephants and alligators were housed in single species enclosures, the latter individually separated by a physical barrier. Giraffes shared their enclosure with zebra, *Equus quagga*, ostrich, *Struthio camelus*, and helmeted guinea fowl, *Numida meleagris*. The emus co-habited with red-necked wallabies, *Macropus rufogriseus* in a walk-through enclosure with restricted visitor access. The 7169 m^2^ elephant enclosure abutted a perimeter fence shared by the zoo with an adjacent park and contained a pool, rocky outcrop heavily planted with trees and a grassed paddock covering over 50% of the area. The 4841 m^2^ giraffe enclosure incorporated two heavily planted rocky outcrops. A raised public walkway and paths surrounded two-thirds of its circumference. The 1536 m^2^ emu/wallaby enclosure incorporated an undulating terrain planted with shrubs and trees. The alligators each occupied a moated enclosure with a total area of 544 m^2^ divided by a raised public walkway.

### 2.3. Experimental Protocol

#### Construction Noise Measurement and Exposure

The audible frequency range for a typical human is generally accepted to be 20 Hz to 20 kHz [31]. Many animal species, from a range of taxa are able to hear and communicate at frequencies far higher than this in the ultrasonic range [32,33,34,35,36], or in the infrasonic range, (those sounds below 20 Hz), the latter including giraffes, ratites (e.g., cassowaries *Casuarius bennetti*), and alligators, among others [4,37,38,39,40]. As these sound frequencies are outside the range of human hearing, auditory equipment is required to detect and analyse them. Humans also perceive the loudness of sounds at different frequencies differently—typically being most sensitive to sounds at frequencies between 1000 Hz and 10 kHz. Different ‘frequency-weighted’ sound pressure levels have been proposed to account for this phenomenon (e.g., the A-weighted sound pressure level). However, no such metric exists for animals and thus, following Owen et al. [11], we used a linear weighting for all sound pressure levels reported in this study.

**Figure 1:**
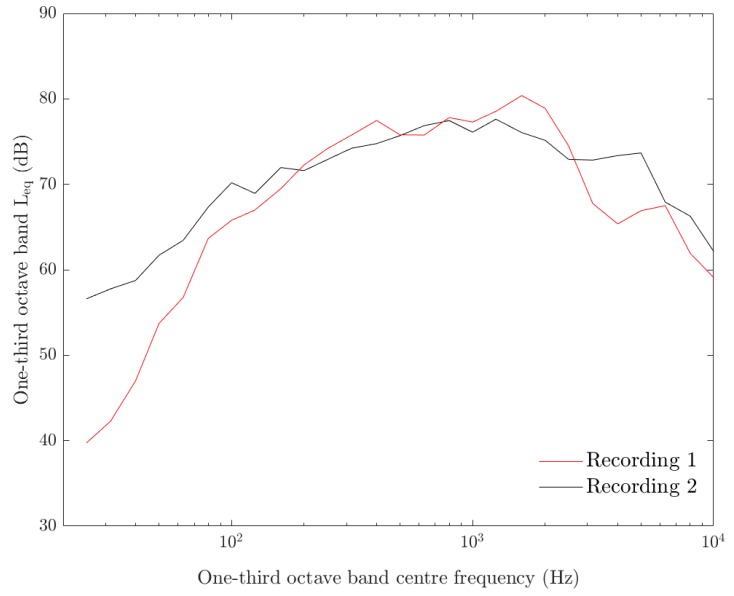
One-third octave band L_eq_ level 1 m in front of the speaker during the continuous noise experiment. Recording 1 (black), recording 2 (red).

Four 90-min recordings of construction noise were created and played through one or two loudspeakers located on the periphery of each animal enclosure. The first recording contained typical truck sounds (engine noise, beeping, loading noise etc.) and other machinery noise (diggers, hand tools), whilst the second recording contained similar noises in addition to impulsive sounds including rock breaking and hammering. These recordings comprised the “Continuous” sound treatments. Each of these files was then re-edited to provide two “Intermittent” sound treatments which included three pauses (where no sound was produced) of between 5 and 10 min within the 90-min period. The one-third octave band L_eq_ level for the two continuous noise recordings measured one meter from the speaker is shown in Figure 1 below.

**Figure 2:**
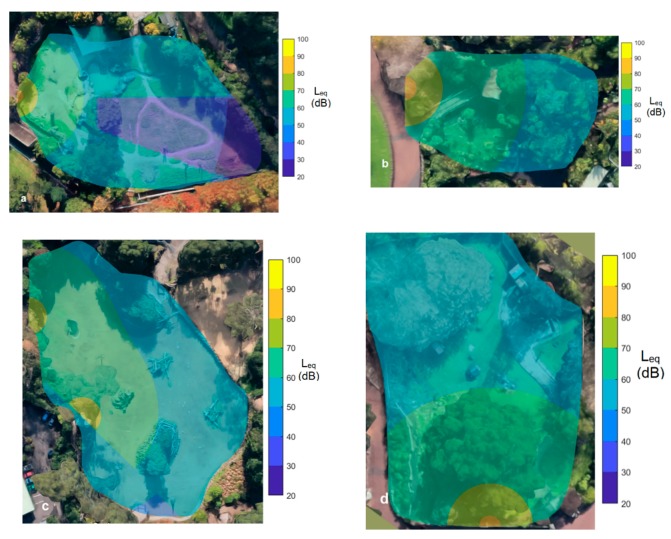
Contours of predicted L_eq_ (dB) at focal species ear heights in the (**a**) elephant, (**b**) alligator, (**c**) giraffe and (**d**) emu enclosure. (Imagery ©2019 Google, MapData Sciences Pty Ltd., PSMA).

The speakers (Fenton, FPS15, 15′′, 350 W portable speaker) were mounted on tripods such that the center of the speaker diaphragm was approximately 1.5 m above the ground and placed in a public space immediately adjacent to, and facing into, the enclosures of the focal animals. Due to differences in area and configuration of the four enclosures, two speakers were stationed outside the giraffe and elephant enclosures and just one speaker outside the emu and alligator enclosures (Figure 2a–d). The L_eq_ level for both construction noise recordings was such that it was at a level of 87 dB, one m directly in front of the speaker whilst construction noise was being produced. Prior to each exposure, the speaker level was calibrated by playing a calibration recording of white noise through the speaker and adjusting the speaker level until the measured L_eq_ noise level one meter directly in front of the speaker diaphragm was 85 dB. Noise measurements were made using a calibrated Class 1.01 dB DUO Smart Noise Monitor with ½” microphone (manufactured by ACOEM Group).

Animals were exposed on different days to one 90-min period of each of the four noise files (continuous recordings 1 and 2; intermittent recordings 1 and 2) and two control periods of the same duration. At least 48 h elapsed between treatments and, to avoid potential conditioning effects, the sequence in which the experimental noises were played were random (Table 1). Ideally, the times of exposure would eliminate the confounding impact of visitors who have access between the hours of 09:30 and 17:30. However, due to animal management routines, this was not feasible for elephants or giraffes but was possible for emus. Alligators were scheduled for late morning as these animals become more responsive to environmental stimuli with rising environmental temperature during the day.

### 2.4. Enclosure Noise Contour Mapping

In order to determine the sound pressure levels within each enclosure during the noise exposure tests, sound pressure level contour maps were generated for each enclosure using the following method: The directivity of the speakers in each one-third octave band was measured by installing the speaker within the anechoic chamber within the Acoustics Laboratories at the University of Auckland. Pink noise was played through the speaker and the sound pressure level in each one-third octave band was measured at a distance 3.5 m from the center of the speaker diaphragm at 10° intervals in a horizontal plane over a 180° arc in front of the speaker. The speaker was assumed to produce noise levels which were axisymmetric about the center of the speaker diaphragm and levels at angles between the measured angles were calculated via linear interpolation. A 3D map of each enclosure was generated from contour maps of the enclosures. This map included the ground coordinates and the gradient of the ground surface which was required for the ground reflection calculations. The L_eq_ sound level at ear height produced by the speaker broadcasting construction noise was calculated using the procedure similar to that described in ISO 9613-2 [41] which included the effects of source directivity, geometrical divergence, ground reflections and atmospheric absorption. Ear heights for the different animals assessed were taken to be: 2.5 m for elephants, 5.5 m for giraffes, 0 m for alligators and 1.5 m for emus. Ground reflections were modelled using the method described in Section 5.9.2 of Bies and Hansen [42] assuming the ground could be modelled as a plane surface with gradient equal to the local ground gradient and where reflected and incident waves add incoherently, except on the surface of the water in the alligator pond where coherent addition was assumed. The large barrier in the elephant enclosure was modelled as providing a 20dB sound reduction at locations out of direct line of sight of the speaker.

The L_eq_ level predictions were validated at 4–6 discrete locations within each enclosure (including in the shadow zone of the large barrier in the elephant enclosure). All measurements were found to be within ±3 dB of the predictions. Contour maps of constant Leq level at ear height and with resolution of 10 dB were then generated for each animal enclosure. These contour maps are presented in Figure 2.

For the alligators, additional acoustic measurements were made underwater using a hydrophone (SoundTrap 300, Ocean Instruments NZ, Warkworth, New Zealand). These measurements showed that at a depth of 0.4 m below the surface of the water, the noise levels with noise generated by the speaker were identical to those present without the speaker playing. This indicates that construction noise levels below the water are negligible.

### 2.5. Behavioral Data Collection

Animal behavior was captured using one to two high-definition digital video cameras (Canon XA30 and Panasonic V770) mounted on tripods. Whenever possible, all focal animals within an enclosure were captured by at least one camera although, on occasion, an animal moved out of sight of the camera view. Cameras were mounted on the periphery of the enclosure for elephants, giraffes and alligators, and were inside the enclosure for the emu recording but within the public path. Continuous 90-min recordings were made throughout control and experimental treatments, and at the same time of day (Table 1). The cameras split the long recordings into several files, therefore files from a single observation period were compiled using Windows Movie Maker and Video Editor (Microsoft Corporation, Redmond, WA, USA). Video files were blind labelled and muted so that video coders were unable to detect which sound treatment the animals were experiencing. Behavior was coded from the videos using CowLog (v.3.02, Natural Resources Institute, Helsinki, Finland, © Matti Pastell matti@cowlog.org) after developing a behavioral catalogue for each species. Catalogues were developed by adapting published ethograms of each species [39,43,44,45,46,47,48] and through consultation with zoo staff (Appendix A). One trained observer coded all of the video for a single species, however observer reliability was checked by a second observer with inter-observer reliability pass criteria being greater than 80% agreement. This required between two and three attempts with training and discussion of behavioral definitions undertaken between each attempt. Inter-observer consistency improved until the required level of agreement was achieved. Behavioral data was coded using focal sampling of two behavior types; the first a set of mutually exclusive long-duration behaviors using continuous recording, and the second a set of short-duration event behaviors using all-occurrence recording. The location of each animal within the soundscape (a map grid reference of 2 m^2^ squares converted to a sound level category using the soundscape map, (Figure 2a–d)) and focal animal proximity to their nearest neighbor (A: within one body length, B: 1–2 body lengths, C: more than 2 body lengths) was also recorded using scan sampling at 5-min intervals. Proximity and location were not recorded for alligators as they were housed singly, and their enclosures had a homogenous soundscape. However, 5-min scans recorded whether they were in or out of the water.

### 2.6. Fecal Collection from Emus

Control fecal samples were collected over four consecutive days beginning 8 August 2017 and experimental samples were collected in the same month subsequent to this. In both cases collection followed the same schedule. At 18:00 on the evening prior to this collection period, all visible feces were removed from the enclosure. Feces were then collected at 07:30, 12:30, 15:30 and 18:00 the following day. During the experimental period, the same fecal-collection routine was followed. On these days, the birds were exposed to recorded construction noise between the hours of 08:00 and 9:30 (Table 1). The birds’ keepers were able to recognize the physical differences in appearance of feces produced by the two birds and all sample collectors were trained to enable them to do this. At each collection, all feces from each bird were scooped up with the clean flat-ended metal blade of a paint scraping tool and placed in a plastic zip-lock bag together with an identification label completed with the bird’s identity, date and time of collection and total wet weight of the sample. To avoid cross-contamination, each bird’s feces were collected with a dedicated, labelled paint scraper. On a few occasions, when it was not possible to distinguish feces from the two birds, a pooled sample was collected and marked as such. After weighing, samples were frozen and transported for analysis to Massey University in Palmerston North.

### 2.7. Fecal Corticosterone Metabolite Measurement in Emus

Fecal samples were prepared in the laboratory following the method used by Fraisse and Cockrem (2006) [49] for chicken fecal samples and by Cockrem et al. (2012) [50] for Japanese quail fecal samples. Fecal samples were freeze-dried for 3 days then weighed, ground, and extracted in ethanol. The mean recovery of corticosterone in extracted fecal samples was 63.7 + 1.0% (n = 15). Individual percentage recovery values were used to calculate results for all the samples. Fecal corticosterone metabolite concentrations were measured in diluted buffer extracts, with fecal metabolite concentrations calculated as nanograms of corticosterone metabolite/gram of dry weight fecal sample. Corticosterone metabolite concentrations were measured by radioimmunoassay following the method of Cockrem et al., 2009 [51] with corticosterone assay reagents from MP Biomedicals (USA). Serial dilutions of diluted buffer extracts in PBSG were parallel to the corticosterone standard curve. The quantitative recovery of corticosterone in fecal extracts was measured by adding different amounts of standard corticosterone to three fecal extracts in PBSG. The recoveries of added corticosterone were 107.9 + 5.5%, 97.0 + 7.0% and 102.9 + 7.5%. The sensitivity of the corticosterone assay was determined as the hormone concentration at the mean minus two standard deviations from the zero hormone point on the standard curves. The assay sensitivity, expressed as ng steroid/g dry weight fecal sample, was 2.45 ng/g. Solutions of corticosterone in PBSG were used as low and high controls in every assay. The intra-assay coefficients of variation were 8.4 and 6.0%, and inter-assay coefficients of variation were 14.6 and 16.6%.

### 2.8. Ethics

Animal use within this study was approved by the Auckland Zoo Ethics Committee, approval number 2017-Z009. Care of the animals in this study was guided by the New Zealand Animal Welfare Act (1999) [52] and the Animal Welfare (Zoos) Code of Welfare (2005) [53]. Animals used in the study were part of the Auckland Zoo collection and remained in the collection after the study, therefore all of the animals’ normal husbandry protocols including enrichment and diet were continued throughout the study. As external enclosures were used during this study, environmental temperature and the light/dark cycle was not manipulated. The ARRIVE guidelines were consulted in the reporting of this research [54].

### 2.9. Data Treatment and Statistical Analysis

Behavioral data were collated, cleaned and checked in Excel (Microsoft Excel for Mac, version 16.23, Microsoft Corporation, Redmond, WA, USA). Descriptive statistics and plots were constructed using R (R Core Team, Vienna, Austria 2009) statistical software with the RStudio interface (RStudio Team, Boston, MA, USA, 2015) and Microsoft Excel. This study focused on animals within the Auckland Zoo collection, and therefore, due to small numbers of individuals per species, formal statistical analysis and generalization outside of the study individuals was generally not possible. There were, however, some behavioral categories that were observed across three of the four species (giraffes, elephants, and emus). These categories were Standing, Feeding, Grooming, Locomotion, Abnormal behavior, and Displacement behavior. Friedman’s tests were conducted on this combined data (n = 7), with Wilcoxon signed-rank tests used for post hoc comparisons with R.

Fecal corticosteroid data were analyzed using Prism (GraphPad Software, LA Jolla, CA, USA). Fecal corticosterone concentrations were log-transformed before analysis. Mean fecal corticosterone concentrations were compared between treatments using one-way ANOVA with post hoc comparisons made with Tukey’s multiple comparisons tests. Data are presented as individual values or as mean ± S.E.

## 3. Results

### 3.1. Acoustic Measurements and Analysis

The predicted L_eq_ noise level contours (10 dB resolution) for each of the enclosures for exposures using the first noise recording are shown in Figure 2. Ambient background noise levels are not incorporated into these plots but were typically between 35 dB and 65 dB in all enclosures.

### 3.2. Animal Behavior

#### 3.2.1. Activity Budgets

**Figure 3:**
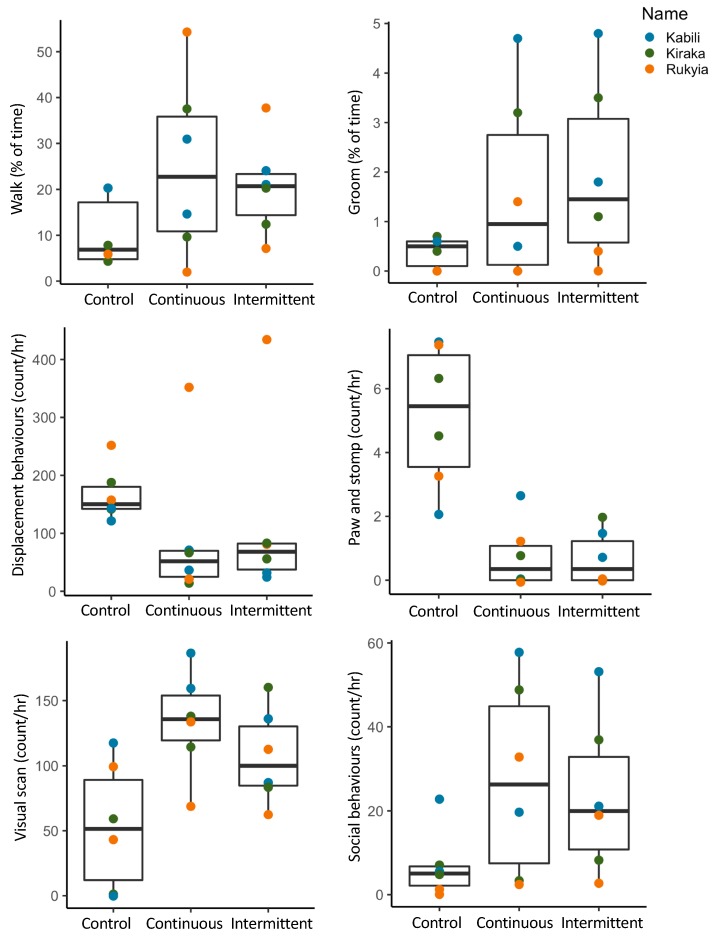
Boxplots of giraffe behavior in control, continuous and intermittent noise conditions, with colors identifying individuals.

Visualization of descriptive statistics from the giraffe behavior suggested several possible behavioral changes due to sound exposure (Figure 3). Four behaviors appeared to increase in response to both continuous and intermittent construction noise. These were walking, grooming, visual scanning of the environment, and social behaviors (Figure 3). Two other behaviors, displacement behaviors (e.g., snorting and head tossing), had distinct observed decreases in occurrence, as did pawing/stomping (Figure 3).

**Figure 4:**
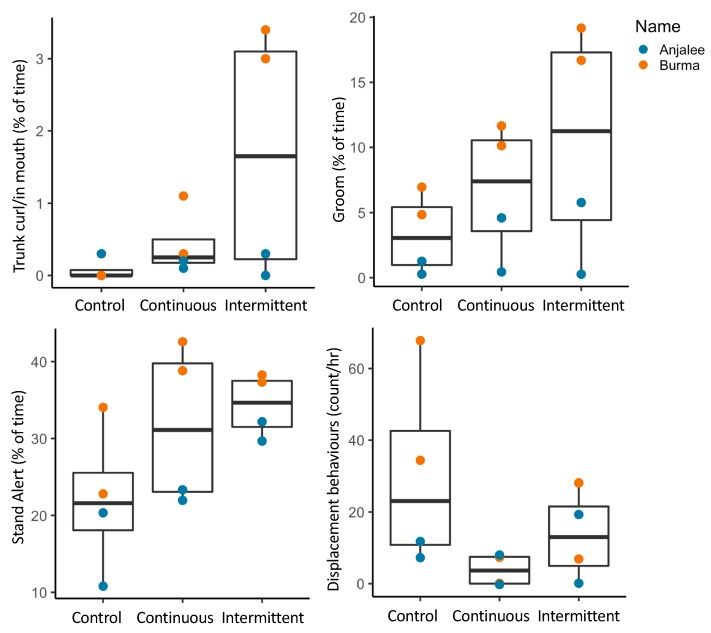
Boxplots of elephant behavior in control, continuous and intermittent noise conditions with symbols identifying individuals.

Descriptive statistics of elephants also suggested behavioral changes in response to sound treatments (Figure 4). Trunk related behaviors (curling, and placement in the mouth), foot lifting, and standing in an alert posture, all appeared to increase, particularly with the intermittent noise condition, although variation between individuals was also apparent (Figure 4). Displacement type behaviors (e.g., ear flapping and yawning) seemed to decrease with exposure to experimental sound (Figure 4).

**Figure 5:**
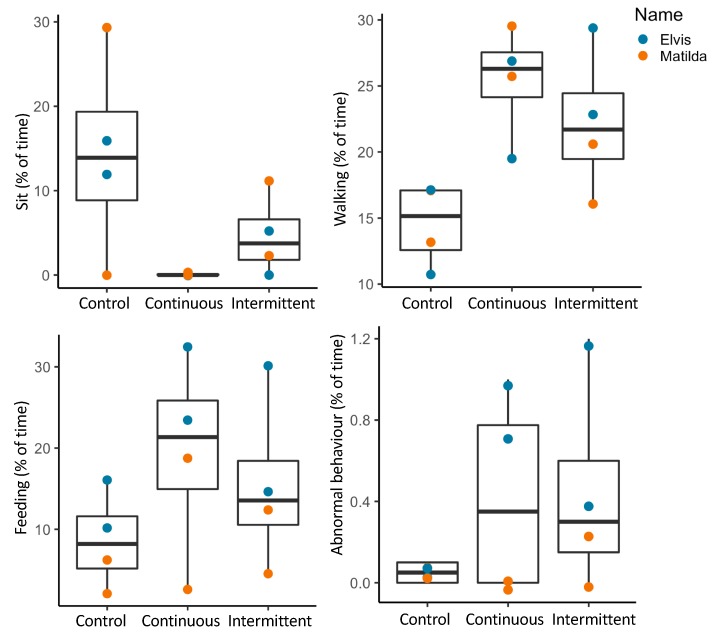
Boxplots of emu behavior in control, continuous and intermittent noise conditions with symbols identifying individuals.

Descriptive statistics of emu behavior indicated that the two emus increased walking, feeding, and abnormal behavior (e.g., fence-pecking) when exposed to construction noise (Figure 5). Sitting behavior had a contrasting pattern. During intermittent noise exposure, sitting behavior was lower than during the control period, and was almost completely absent during continuous noise exposure (Figure 5).

**Figure 6:**
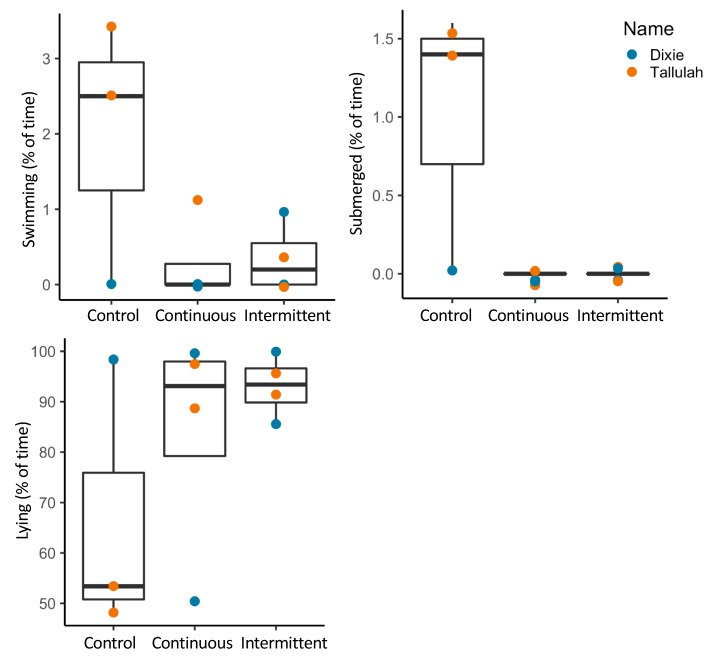
Boxplots of alligator behavior in control, continuous and intermittent noise conditions with symbols identifying individuals.

Visualization of alligator behavior during exposure to experimental sound suggested some variation between the two individuals, however at a species level, time spent fully submerged or swimming appeared to decrease, while lying behavior had a contrasting pattern (Figure 6).

#### 3.2.2. Combined Behavioral Categories

Several behavioral categories were statistically analyzed using data combined from giraffes, elephants and emus. Locomotion behavior was significantly higher during continual exposure to construction noise than when exposed intermittently or during the control condition (Table 2). Displacement behavior had a statistical trend, but post hoc comparisons between sound categories were all non-significant (Table 2).

### 3.3. Location Changes and Social Proximity in Response to Sound

**Figure 7:**
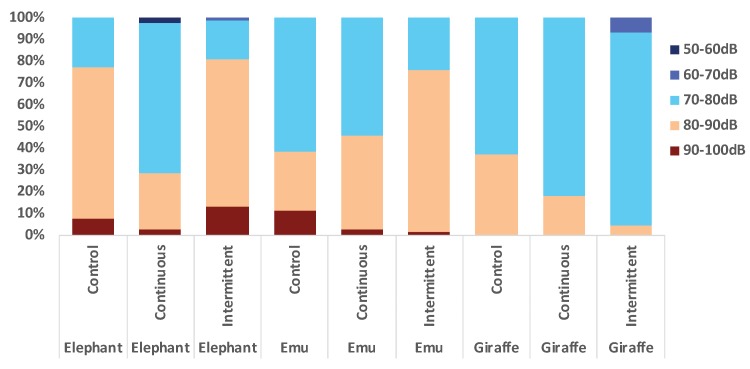
Time spent in enclosure locations during control, continuous, and intermittent sound conditions (mean % of time).

**Figure 8:**
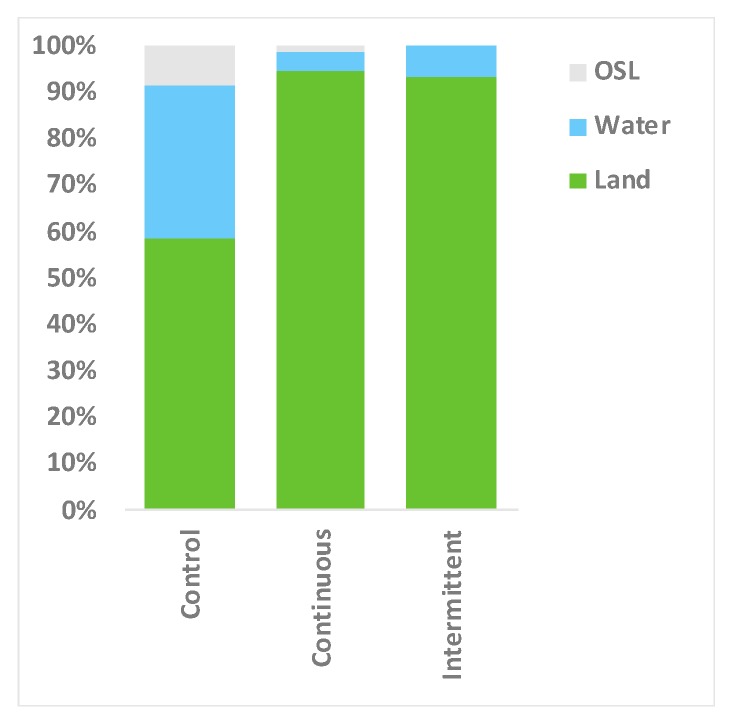
Time spent in water and on land by each alligator (mean % of time) in control, continuous and intermittent noise conditions. (OSL = Out of Sight).

**Figure 9:**
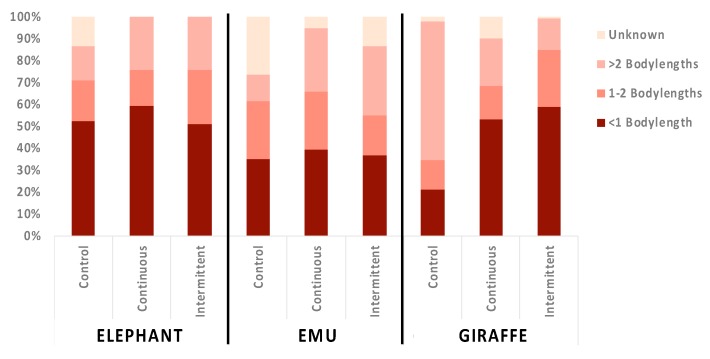
Time spent in proximity to the nearest conspecific during control, continuous, and intermittent sound conditions (mean % of time).

Location data from the two elephants indicated that they moved into quieter areas when exposed to continuous but not intermittent construction noise (Figure 7). The giraffes also moved to quieter enclosure locations during noise exposure, but this was most evident during the intermittent noise (Figure 7). Emus decreased the time spent in the loudest and quietest locations, increasing their time in the area with a mid-range volume during sound exposure (Figure 7). The amount of time spent in and out of water by the alligators was not obviously influenced by noise exposure (Figure 8). The amount of time that elephants and emus spent in close proximity to each other was not conspicuously affected by sound exposure (Figure 9). In contrast, giraffes spent more than twice as much time in close proximity to a conspecific during construction noise exposure, than during the control period (Figure 9).

### 3.4. Emu Fecal Corticosterone

**Figure 10:**
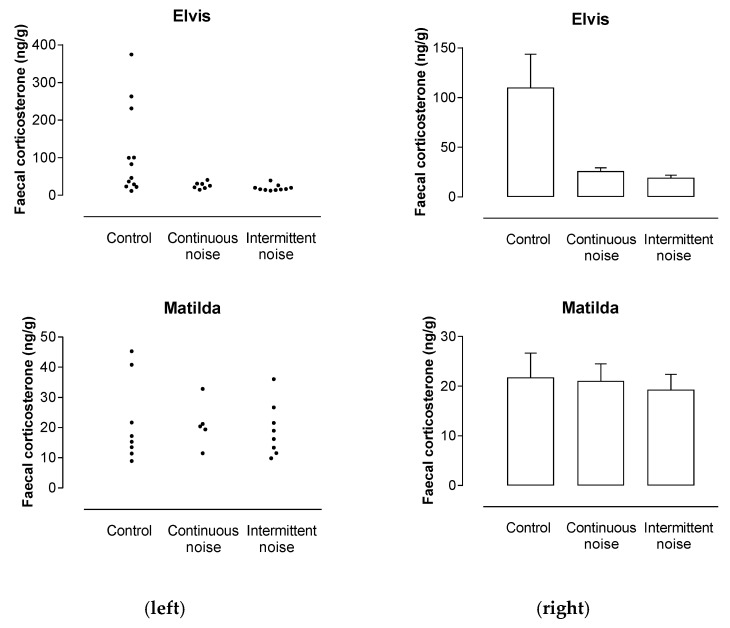
Fecal corticosterone metabolite concentrations in individual samples (**left**) and mean fecal corticosterone metabolite concentrations (**right**) in samples from Elvis and Matilda.

Individual fecal corticosterone metabolite concentrations are shown for emus Elvis and Matilda in Figure 10. Concentrations ranged from 11.6 to 374.7 ng/g in control samples from Elvis and from 8.9 to 45.3 ng/g in control samples from Matilda. Concentrations ranged from 12.6 to 41.0 ng/g in treatment samples from Elvis and from 9.8 to 36.1 ng/g in treatment samples from Matilda.

One-way ANOVAs showed that there were significant differences between collection periods in mean fecal corticosterone metabolite concentrations in samples from Elvis, and no significant differences in samples from Matilda (*F*_2,26_ = 8.282, *p* = 0.002; F_2,18_ = 0.0935, *p* = 0.911 respectively). Mean corticosterone metabolite concentrations (Figure 10) were greater in control samples than in continuous or intermittent treatment samples from Elvis (*p* < 0.05) and did not differ between continuous and intermittent treatments in Matilda (*p* < 0.05).

## 4. Discussion

In this study, four different species were exposed to three noise types: Control (ambient conditions), continuous construction and intermittent construction noise. While the small sample size restricted the amount of formal statistical testing that could be performed, descriptive statistics of activity budgets appeared to demonstrate changes in all species occurring as a result of sound exposure, with variation occurring between species, individuals and sound types. Enclosure use and social spacing also appeared to change with noise exposure, but patterns were not consistent between species.

In this study, giraffe behaviors that appeared to change in response to experimental construction noise included locomotion, vigilance, grooming and social behavior. These behaviors can be indicative of agitation and threat detection [55] and in the context of captive animal welfare, may be symptomatic of negative affective states (e.g., [2,56]). Previous wild giraffe studies show increases in vigilance in response to close proximity of a conspecific, particularly bulls [57]. In the current study, social interaction increased, as did the time spent in close proximity when exposed to construction noise. Giraffes are social animals with a fission-fusion social structure that fluctuates in response to context and strengthens with external threats such as habitat disturbance [58]. Perceived external threats may also increase proximity, especially between a calf and mother [59]. During the course of this study, the composition of the giraffe herd changed following initial control observations and prior to the experimental treatments with the death of one aged animal and the inter-zoo transfer of another. Consequently, the control data for the remaining animals was obtained at a later date (Table 1). Over the course of this time, the calf aged from 2–8 months, however, giraffe calves are suckled from 9–24 months [60] and, consistent with this, the dam’s level of attentiveness towards her offspring remained high throughout the study period. It is possible that the increase in vigilance was due to a perceived threat from the sound treatments, or from the closer social proximity as found in wild studies [57]. The giraffes in this study also seemed to move to quieter regions of their enclosure during noisy conditions. This is important for two reasons—firstly, it may indicate a preference for quieter locations, which can help to understand how captive animals rank or value aspects of their environment [61]. Secondly, the ability for an animal to have control and choice over their environment is also a key tenet in animal welfare science [62]. The behavioral changes exhibited by the giraffes suggest that they found exposure to construction noise aversive. However, displacement type behaviors and pawing/stomping behaviors appeared to be higher during the control condition. Displacement behaviors such as yawning or head shaking are often self-directed and appear irrelevant in the context in which they are performed [63,64]. They are often performed in negatively perceived contexts, however in the current study they were actually performed less during the experimental treatment, and a similar pattern was noted for elephants. It may be that this discrepancy is because they did not find sound exposure particularly aversive, however displacement behaviors are frequently associated with social anxiety (e.g., [63,65,66]). With sound exposure, the giraffes appeared to decrease the distance between conspecifics and it may therefore be that displacement behavior is not a strong indicator of environmental stress in these animals but further research would be needed to draw a conclusion.

Elephants displayed some of the same behavioral changes as the giraffes. During exposure to continuous sound, they seemed to move into quieter areas of their enclosure, and perform more standing alert behavior (indicative of vigilance), trunk-related behaviors and foot lifting, although these appeared more pronounced for intermittent noise (Figure 4). Trunk curling is thought to be a behavior that occurs when elephants feel uneasy or apprehensive [67], while vigilance behavior also suggests a heightened perception of threat or a feeling of alarm [67,68] and foot lifting-type actions may be a type of displacement behavior [67,69] (i.e., a behavior that is seemingly out of context but may indicate apprehension or conflicting motivations), although other displacement behavior showed a decreasing pattern. Elephant feet are also key sensory body parts for the detection of low frequency sound, which is used in vocal communication [68] and may therefore be sensitive to non-communicative vibrational changes. As for the giraffes, exposure to construction noise appeared to be aversive to the elephants in this study.

The emus in the study demonstrated behavioral changes to construction noise that were suggestive of an aversive nature but few studies have focused on the behavior and welfare of this species. We found emus increased their ingestive behavior and locomotion, and decreased sitting. Significantly increased locomotion was a general response found across three of the four species. A shift from resting to locomotion behavior may indicate increased agitation, but it would be anticipated that feeding behavior would also decrease. A review on ratite welfare suggests that stress behaviors tend to present as stereotypies, self-directed behavior, panicked behavior, and agonism [45]. Abnormal behaviors were presented at very low rates in these animals although they did appear to increase with sound exposure. When construction noise was played, emus moved away from the area closest to the speaker which had the loudest corresponding sound level. During this time, they spent more time in the mid-volume regions of their enclosure, however this is difficult to interpret in the context of aversion to sound.

Mean fecal glucocorticoid concentrations did not differ between control and noise treatment periods in one emu, whereas concentrations were more than five times higher in the control than the treatment periods in the second emu (Elvis). Mating was observed on two of the days in the control period and one of the days in the treatment period. The elevated glucocorticoid concentrations in the male may have been associated with mating and these elevated concentrations confound the interpretation of the results for this bird. However, glucocorticoid concentrations in both birds were similar on the noise treatment days and the results together indicate that the emus did not perceive the sound treatment to be a stressor. Concurrent behavioral observations suggest that, at the volumes and duration experimental sound was projected, emus may have found the experience mildly aversive, but a strong reaction that would cause concern for ongoing welfare, was not evident.

Alligators did demonstrate behavioral changes with sound, however these were hard to interpret for several reasons. Firstly, we were unable to find any published studies on how alligators respond behaviorally to noise or stress. Secondly, the alligators spent long periods of time in the same position/location/behavior, which means that their responses may not be directly due to sound but may be incidental. Behaviors that appeared to change with sound were swimming and being fully submerged, which both appeared to decrease but did not occur often, even in the control conditions. Lying behavior out of the water seemed to increase considerably from the control condition to when they were exposed to construction noise however individual variation was apparent. Social spacing behavior was not recorded as the alligators were housed singly, and location data was restricted to “in water” or “out of water” as the sound environment out of the water was homogenous. Interestingly, our hydrophone data suggested that construction noise almost entirely disappeared underneath the water. However, despite this, the alligators did not appear to increase their in-water behavior with exposure to sound. Therefore, an aversive response to noise was not evident for this species in this limited study but the possible use of water as a potential refuge from noise by this species is worthy of further research. We suggest, in order to preserve welfare, that a conservative approach be taken in applying our results to real life scenarios in case longer observation periods, alternative measures, and a larger sample size produce a clearer picture of responses to noise from this species.

## 5. Conclusions

The experimental period used in this study was short—90 min—in contrast to the length of exposure time in a real-life construction situation. It is possible that animals may habituate over time to noise of this type, however we would caution that this would be a significant assumption, as habituation does not always occur to aversive situations and, when animals have no option to remove themselves from the situation they may develop ‘learned helplessness’—a disorder characterized by loss of behavioral response but continued physiological and emotional response [70]. Overall, our behavioral results, in particular from giraffes and elephants, indicate that exposure to construction noise may be an aversive experience for many animals, and precautions should be taken by zoos to minimize the duration and intensity of exposure, when elimination is not a feasible option.

Informed, in part, by this study, Auckland Zoo established protocols, prior to the commencement of construcion in 2019, to mitigate the potential impacts of construction noise on its animals. These included avenues for continuous liaison between contractors and keepers, advance warning of particularly noisy work (e.g., rock breaking) and continuous liaison during works to enable an immediate halt to activities should any animal show significant indications of stress. Specific mitigating actions varied according to species but have included one or more of the following as deemed appropriate:

1. Scheduling the work to occur while animals were closely monitored by keepers.
2. Keeper review of closed-circuit overnight video recordings to monitor elephant behaviour including sleep patterns.
3. Timing specific construction activities to fit in with animal activity periods or behavioural cycles.
4. Providing distraction for the animals via, for example, training activities (elephants) or provision of enrichment opportunities.
5. Modifying routine husbandry procedures to allow individuals access to additonal or alternative parts of their enclosures.
6. The application of a commercial sound-absorbant material (Hushtec^®^ Acoustic Exterior Curtain, Duraflex Distribution) to construction site fencing adjacent to the emu enclosure.

## Figures and Tables

**Table 1:** Schedule of noise exposure treatments: Control (ambient sound), Continuous construction noise (Contin), and Intermittent construction noise (Inter). All recordings were collected in 2017 except for the Giraffe Control treatments which were in 2018.

Species (N)	Time	Control 1	Control 2	Contin 1	Contin 2	Inter 1	Inter 2
**Giraffe (3)**	09:45–11:15	15-Feb	22-Feb	21-Aug	23-Aug	28-Aug	7-Sep
**Alligator (2)**	11:30–13:00	7-Feb, 14-Feb	27-Feb	20-Sep	13-Sep	5-Sep	19-Sep
**Emu (2)**	08:00–09:30	7-Feb, 21-Feb	14-Feb	15-Aug	17-Aug	22-Aug	24-Aug
**Elephant (2)**	09:30–11:00	25-Jul	26-Jul	16-Aug	1-Sep	25-Aug	29-Aug

**Table 2:** Mean ± SE of behavioral categories from giraffes (n = 3), elephants (n = 2), and emus (n = 2) during three sound conditions (control, continuous exposure to construction noise, intermittent exposure to construction noise). All means are % of time, except for displacement behavior which is rate/hr. Statistically significant differences between groups (using Wilcoxon signed-rank tests) are indicated by superscript (Different letters indicate *p* < 0.05).

Behavior	Control	Continuous	Intermittent	Friedman’s Tests
Standing (%)	42.6 ± 7.7	48.7 ± 5.8	36.8 ± 3.6	χ^2^ = 2, df = 2, *p* = 0.37
Feeding (%)	25.9 ± 6.3	16.9 ± 3.8	28.3 ± 4.9	χ^2^ = 0.9, df = 2, *p* = 0.65
Grooming (%)	3.3 ± 1.6	3.9 ± 0.9	4.6 ± 1.3	χ^2^ = 3.7, df = 2, *p* = 0.16
Locomotion (%)	13.5 ± 2.2 ^a^	23.5 ± 1.4 ^b^	18.2 ± 2.3 ^a^	χ^2^ = 8.9, df = 2, *p* = 0.01
Abnormal (%)	0.23 ± 0.21	0.5 ± 0.3	0.3 ± 0.1	χ^2^ = 0.7, df = 2, *p* = 0.69
Displacement (rate/hr)	86.0 ± 30.3	44.15 ± 24.8	62.7 ± 33.3	χ^2^ = 5.4, df = 2, *p* = 0.07

**Figure A1:**
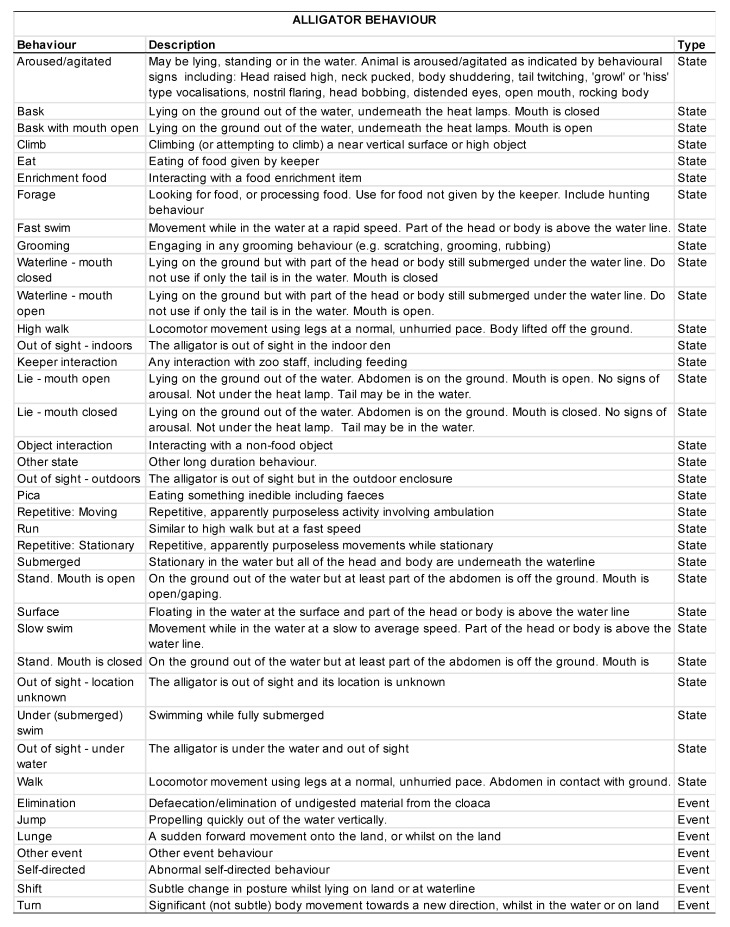

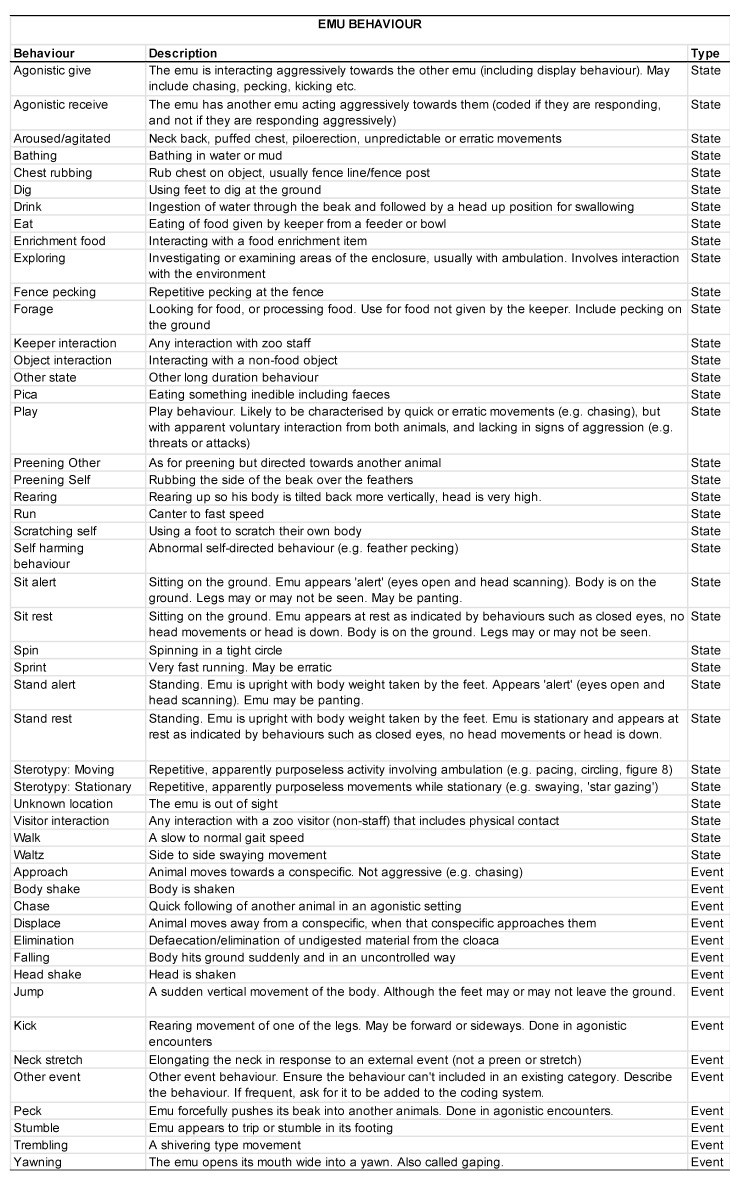

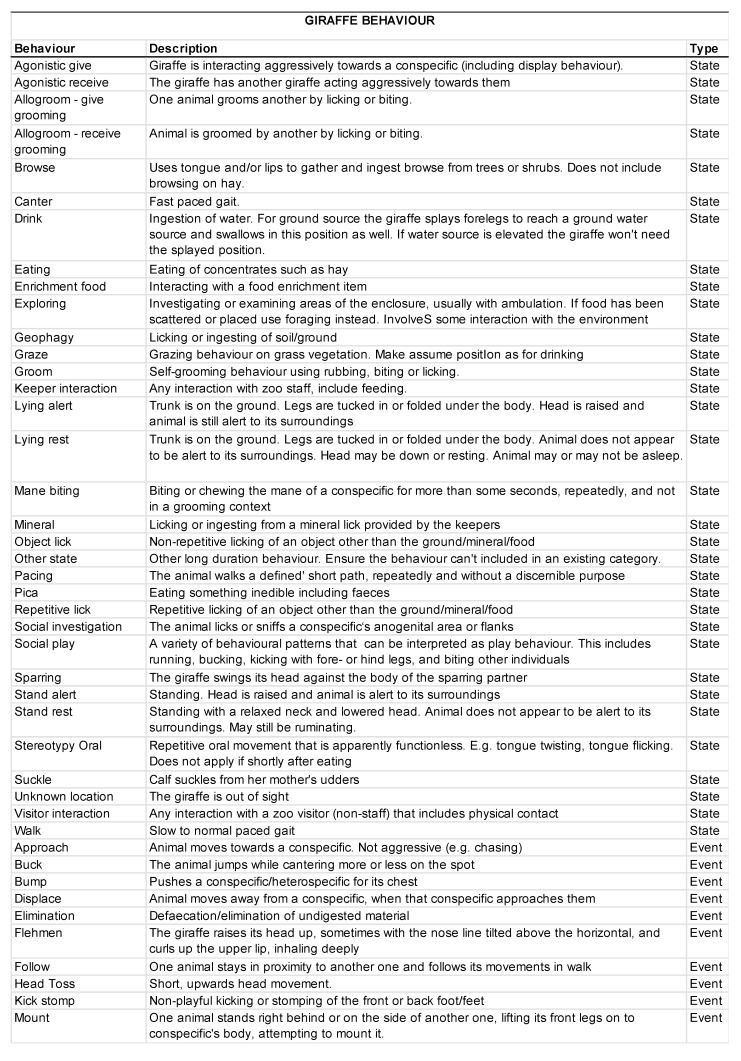

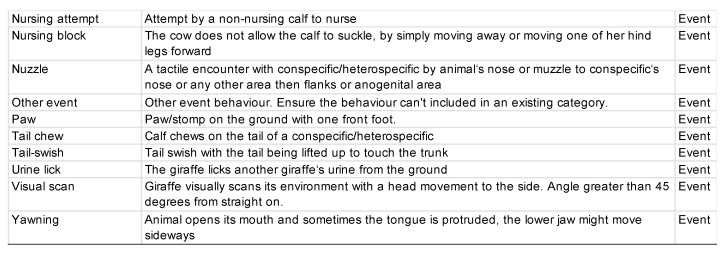

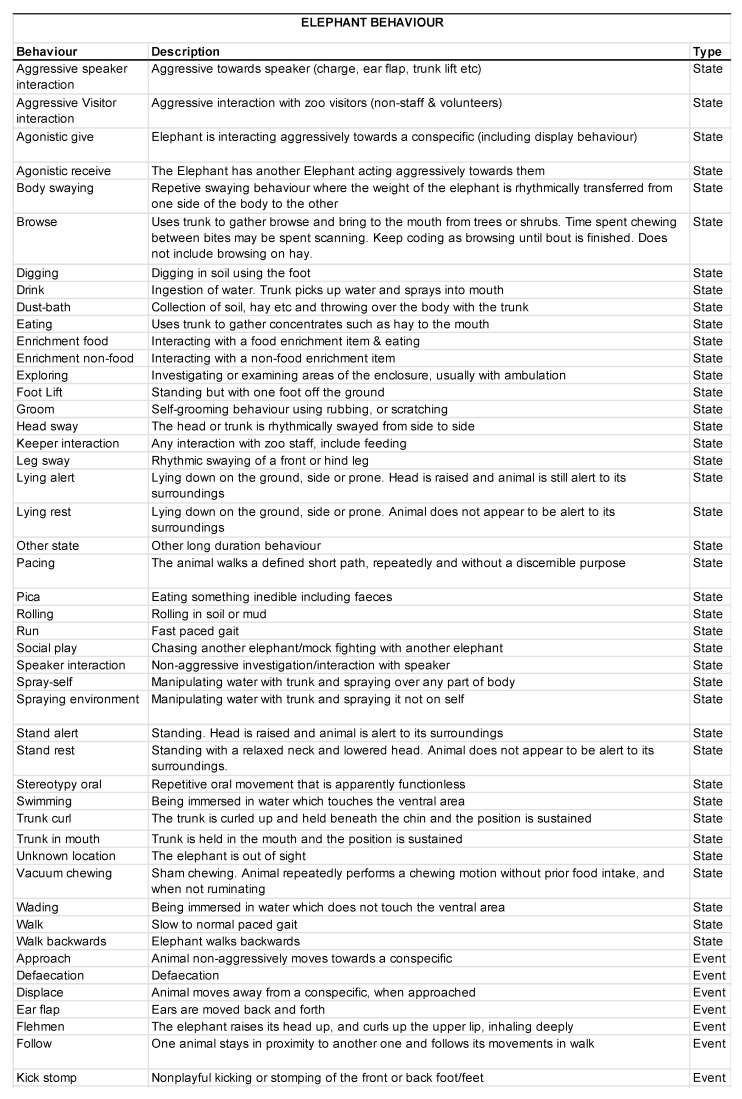

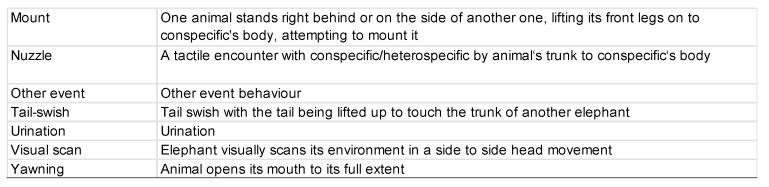
Ethograms used to code behavioral observations of focal animals.
